# Supplementary material for: Scoping Review: Methods and Applications of Spatial Transcriptomics in Tumor Research
Source: Cancers (Basel). 2024 Sep 6;16(17):3100. doi: 10.3390/cancers16173100 (PMC11394603; doi:10.3390/cancers16173100)
Supplement: Supplementary file 1 [file cancers-16-03100-s001.zip › cancers-3140520-supplementary.pdf]

Review

Scoping Review: Methods and Applications of Spatial Transcriptomics in Tumor Research

Kacper Maciejewski [1,*] and Patrycja Czerwinska [1,2,3]

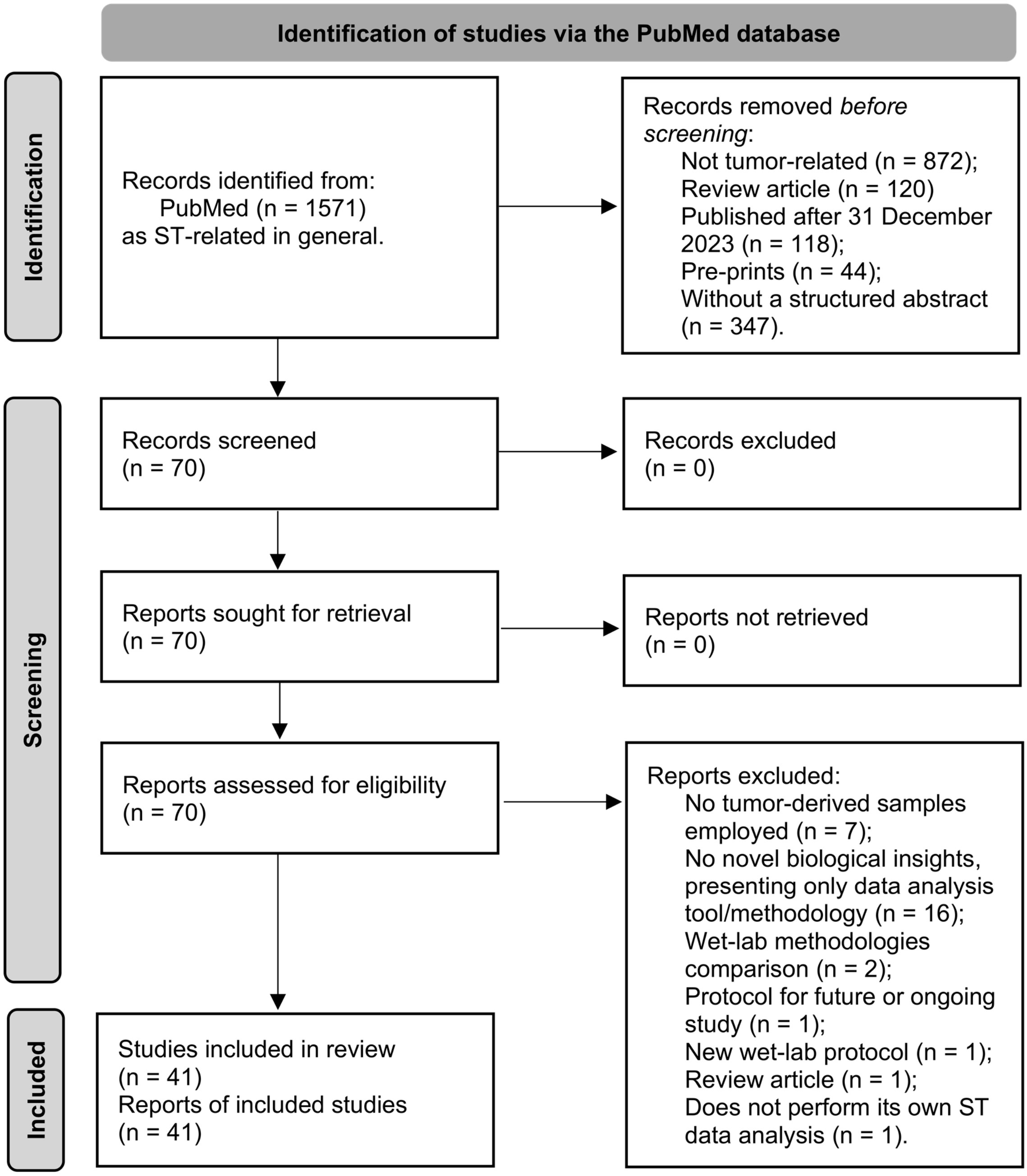

Figure S1. The selection of evidence sources workflow for the scoping analysis, in accordance to the PRISMA-ScR guidelines. The flow diagram structure based on Tricco et al. [24].

Table S1. Individual queries applied to GEO (Gene Expression Omnibus) and PubMed repositories to estimate current trends in spatial transcriptomics tumor research. Cancer-specific tags in each query (bolded) have been built upon manual inspection of available samples in the GEO repository for spatial transcriptomics in general.

Repository	Organ	Query
GEO	pancreas	(spatial transcriptomics) AND (tumor OR tumour OR neoplasm OR cancer OR carcinoma) AND "Homo sapiens"[porgn:__txid9606] AND ("0001/01/01″ : "2023/12/31") AND **(pancretic OR pancreas OR pdac)**
	breast	(spatial transcriptomics) AND (tumor OR tumour OR neoplasm OR cancer OR carcinoma) AND "Homo sapiens"[porgn:__txid9606] AND ("0001/01/01″ : "2023/12/31") AND **(breast OR tnbc)**
	liver	(spatial transcriptomics) AND (tumor OR tumour OR neoplasm OR cancer OR carcinoma) AND "Homo sapiens"[porgn:__txid9606] AND ("0001/01/01″ : "2023/12/31") AND **liver**
	brain	(spatial transcriptomics) AND (tumor OR tumour OR neoplasm OR cancer OR carcinoma) AND "Homo sapiens"[porgn:__txid9606] AND ("0001/01/01″ : "2023/12/31") AND **(glioma OR dmg OR glioblastoma OR gbm)**
	kidney	(spatial transcriptomics) AND (tumor OR tumour OR neoplasm OR cancer OR carcinoma) AND "Homo sapiens"[porgn:__txid9606] AND ("0001/01/01″ : "2023/12/31") AND **kidney**
	lung	(spatial transcriptomics) AND (tumor OR tumour OR neoplasm OR cancer OR carcinoma) AND "Homo sapiens"[porgn:__txid9606] AND ("0001/01/01″ : "2023/12/31") AND **lung**
	lymph	(spatial transcriptomics) AND (tumor OR tumour OR neoplasm OR cancer OR carcinoma) AND "Homo sapiens"[porgn:__txid9606] AND ("0001/01/01″ : "2023/12/31") AND **(lymphoma OR NHL OR lymph node)**
	organoid / xenograph	(spatial transcriptomics) AND (tumor OR tumour OR neoplasm OR cancer OR carcinoma) AND "Homo sapiens"[porgn:__txid9606] AND ("0001/01/01″ : "2023/12/31") AND **(organoid OR xenograph)**
	spleen	(spatial transcriptomics) AND (tumor OR tumour OR neoplasm OR cancer OR carcinoma) AND "Homo sapiens"[porgn:__txid9606] AND ("0001/01/01″ : "2023/12/31") AND **spleen**
	blood	(spatial transcriptomics) AND (tumor OR tumour OR neoplasm OR cancer OR carcinoma) AND "Homo sapiens"[porgn:__txid9606] AND ("0001/01/01″ : "2023/12/31") AND **aml**
	buccal cavity	(spatial transcriptomics) AND (tumor OR tumour OR neoplasm OR cancer OR carcinoma) AND "Homo sapiens"[porgn:__txid9606] AND ("0001/01/01″ : "2023/12/31") AND **(buccal OR oral)**

	bladder	(spatial transcriptomics) AND (tumor OR tumour OR neoplasm OR cancer OR carcinoma) AND "Homo sapiens"[porgn:__txid9606] AND ("0001/01/01″ : "2023/12/31") AND **bladder**
	gastric stroma	(spatial transcriptomics) AND (tumor OR tumour OR neoplasm OR cancer OR carcinoma) AND "Homo sapiens"[porgn:__txid9606] AND ("0001/01/01″ : "2023/12/31") AND **gastric**
	endometrium	(spatial transcriptomics) AND (tumor OR tumour OR neoplasm OR cancer OR carcinoma) AND "Homo sapiens"[porgn:__txid9606] AND ("0001/01/01″ : "2023/12/31") AND **endometrial**
	ovary	(spatial transcriptomics) AND (tumor OR tumour OR neoplasm OR cancer OR carcinoma) AND "Homo sapiens"[porgn:__txid9606] AND ("0001/01/01″ : "2023/12/31") AND **ovarian**
	breast	(spatial transcriptomics) AND (tumor OR tumour OR neoplasm OR cancer OR carcinoma)] AND **(breast OR tnbc)** AND ("0001/01/01″ : "2023/12/31"
**PubMed**	liver	(spatial transcriptomics) AND (tumor OR tumour OR neoplasm OR cancer OR carcinoma) AND **liver** AND ("0001/01/01″ : "2023/12/31"
	pancreas	(spatial transcriptomics) AND (tumor OR tumour OR neoplasm OR cancer OR carcinoma) AND **(pancretic OR pancreas OR pdac)** AND ("0001/01/01″ : "2023/12/31"

Table S2. The full query applied to the PubMed repository to perform a scoping analysis of spatial transcriptomics tumor research methods and applications.

Repository	Query
**PubMed**	"spatial transcriptomics" AND ("cancer" OR "tumor" OR "tumour") NOT "Review"[Publication Type] AND ("0001/01/01"[Date - Publication] : "2023/12/31"[Date - Publication]) AND "english"[Language] AND hasstructuredabstract NOT "preprint"[Publication Type]

Table S3. The list of rejected articles with our reasonings. All the other articles indexed by the query presented in Table S2 were included in the analysis. Due to the fixed time limitation in the query, the resulting list of articles of the PubMed search is constant.

Rejection Reason	PMIDs
the study employed only samples which are not tumor-derived	36029799; 37605313; 37724990; 37019821; 36950091; 37691918; 35985456
the study focuses entirely on presenting new data analysis tools and does not emphasize any novel biological insights	36707753; 37543577; 35258565; 32753032; 36179087; 35941549; 37039825; 36699413; 34358288; 34864909; 37738898; 37285319; 37698995; 37468850; 37756699; 35043939
the study compares different wet-lab methodology protocols for ST sample preparation	36882687; 35792866
the article is a protocol for future or ongoing studies	38066522
the article presents a novel wet-lab protocol for ST sample handling	32293264
the study uses already-analyzed visualization data from the SpatialDB repository	36899366
the article is a review	31871830

Table S4. Overview of selected articles including platforms, tools and methods employed to process ST data. *NA* represents data which was not found for specific articles. *PD* (*public dataset*) means that the original samples were not analyzed. All references are included with the main article text.

PMID	Article Title	Tumor	ST Platform	ST Analysis Tools	ST Analysis Methods	Other Data Types	Ref.
36708811	Identification of a tumour immune barrier in the HCC microenvironment that determines the efficacy of immunotherapy	hepatocellular carcinoma	Visium	SpaceRanger; Seurat; BayesSpace; NicheNet; CellChat	BayesSpace-spatialEnhance; Seurat-FindClusters; Seurat-AddModuleScore; Seurat-SpatialFeaturePlot; Seurat-FindMarkers; Seurat-FindAllMarkers	single-cell; immunohistochemistry; cell culture	[49]
36162205	Integrating microarray-based spatial transcriptomics and single-cell RNA-sequencing reveals tissue architecture in esophageal squamous cell carcinoma	esophageal squamous cell carcinoma	Visium	CellSpace; Seurat; monocle2; survival; ReactomeGSA	Seurat-FindAllMarkers; Seurat-FindTransferAnchors; Seurat-FindMarkers; survival-coxph	single-cell	[53]
36967539	Spatiotemporally deciphering the mysterious mechanism of persistent HPV-induced malignant transition and immune remodelling from HPV-infected normal cervix, precancer to cervical cancer: Integrating single-cell RNA-sequencing and spatial transcriptome	cervical cancer	Visium	SpaceRanger; Seurat; SPOTlight	Seurat-FindMarkers	single-cell	[75]
37081869	Integrating single-cell RNA-seq and spatial transcriptomics reveals MDK-NCL dependent immunosuppressive environment in endometrial carcinoma	endometrial cancer	PD	Seurat; CellMarker; NICHES	Seurat-FindMarkers; Seurat-FindTransferAnchors; Seurat-TransferData	single-cell; bulk-seq	[118]
35794563	Spatial transcriptomics atlas reveals the crosstalk between cancer-associated fibroblasts and tumor microenvironment components in colorectal cancer	colorectal cancer	PD	Seurat; clusterProfiler; monocle2	Seurat-FindClusters; Seurat-SelectIntegrationFeatures; Seurat-FindIntegrationAnchors; Seurat-IntegrateData; Seurat-FindNeighbors; Seurat-FindAllMarkers	bulk-seq	[119]
37263303	Spatially Resolved Multi-Omics Single-Cell Analyses Inform Mechanisms of Immune Dysfunction in Pancreatic Cancer	pancreatic ductal adenocarcinoma	Visium	SpaceRanger; Seurat; Harmony; SpotClean; SingleR	Seurat-FindClusters; Nearest-Neighbor clustering	single-cell; immunohistochemistry; mass spectrometry; cell culture	[67]
37488671	Integrated single-cell and spatial transcriptomic profiling reveals higher intratumour heterogeneity and epithelial-fibroblast interactions in recurrent bladder cancer	bladder cancer	Visium	SpaceRanger; Seurat; SPOTlight; BayesSpace; Giotto; NicheNet; CellTrek	Seurat-FindClusters; Seurat-FindIntegrationAnchors; Seurat-IntegrateData; Seurat-FindTransferAnchors; Seurat-TransferData; Giotto-cellProximityEnrichment; Giotto-spatCellCellcom	single-cell; immunohistochemistry	[64]

36153593	Intratumor heterogeneity and T cell exhaustion in primary CNS lymphoma	central nervous system lymphoma	Visium	SpaceRanger; Seurat	Seurat-FindTransferAnchors	single-cell; flow cytometry	[97]
35618288	Spatial transcriptomics of macrophage infiltration in non-small cell lung cancer reveals determinants of sensitivity and resistance to anti-PD1/PD-L1 antibodies	non-small cell lung cancer	GeoMx	AtoMX; limma	limma-DGE	immunohistochemistry	[134]
37125608	Primary Aldosteronism: Spatial Multiomics Mapping of Genotype-Dependent Heterogeneity and Tumor Expansion of Aldosterone-Producing Adenomas	adrenocortical adenoma	Visium	SpaceRanger; Seurat; gprofiler2; STUtility; cNMF; Clustree; PROGENy	NA	mass spectrometry; immunohistochemistry	[137]
37344903	Interactions between MFAP5 + fibroblasts and tumor-infiltrating myeloid cells shape the malignant microenvironment of colorectal cancer	colorectal cancer	PD	Seurat; CellChat	Seurat-SelectIntegrationFeatures; Seurat-PrepSCTIntegration; Seurat-FindIntegrationAnchors; Seurat-IntegrateData; Seurat-SpatialFeaturePlot; Seurat-SpatialDimPlot; Seurat-AddModuleScore	single-cell; bulk-seq; immunohistochemistry	[133]
37723590	Spatial transcriptomics analysis of neoadjuvant cabozantinib and nivolumab in advanced hepatocellular carcinoma identifies independent mechanisms of resistance and recurrence	hepatocellular carcinoma	Visium	SpaceRanger; Seurat; Harmony; DESeq2; SCENIC; Domino	Leiden clustering	single-cell; bulk-seq	[60]
38115703	Single-cell and spatial transcriptomics reveal POSTN(+) cancer-associated fibroblasts correlated with immune suppression and tumour progression in non-small cell lung cancer	non-small cell lung cancer	Stereo-seq	Seurat; spacexr	Seurat-FindMarkers	single-cell; immunohistochemistry	[38]
37153569	Dissecting order amidst chaos of programmed cell deaths: construction of a diagnostic model for KIRC using transcriptomic information in blood-derived exosomes and single-cell multi-omics data in tumor microenvironment	klidney renal clear cell carcinoma	PD	Seurat; clusterProfiler	NA	single-cell; bulk-seq	[25]
37951222	Artificial intelligence-based pathology as a biomarker of sensitivity to atezolizumab-bevacizumab in patients with hepatocellular carcinoma: a multicentre retrospective study	hepatocellular carcinoma	Visium	SpaceRanger; Seurat	NA	bulk-seq; single-cell	[26]
38077210	Spatial architecture of high-grade glioma reveals tumor heterogeneity within distinct domains	glioblastoma	GeoMx; Visium (PD)	StandR; Seurat; limma; edgeR; cnvkit; spatialDecon; speckle	limma-voom	single-cell	[88]

37124494	Spatial transcriptomics reveals the heterogeneity and FGG+CRP+ inflammatory cancer-associated fibroblasts replace islets in pancreatic ductal adenocarcinoma	pancreatic ductal adenocarcinoma	Visium	Seurat; ST-Pipeline; clusterProfiler; STUtility; Metascape	Seurat-AddModuleScore; Nearest-Neighbor clustering; Seurat-SpatialFeaturePlot	immunohistochemistry	[58]
38050079	Integrative multiomics enhancer activity profiling identifies therapeutic vulnerabilities in cholangiocarcinoma of different etiologies	cholangiocarcinoma	Visium	SpaceRanger; BayesSpace; VarTrix; Seurat; MCP-counter	NA	immunohistochemistry; single-cell; cell culture	[95]
37397378	Integration analysis of single-cell and spatial transcriptomics reveal the cellular heterogeneity landscape in glioblastoma and establish a polygenic risk model	glioblastoma	PD	Seurat; CellTrek; CellChat; monocle2; GeneSwitches	Seurat-FindMarkers; Seurat-FindNeighbors; Seurat-FindClusters; CellTrek-SColoc; CellChat-AggregateNet	bulk-seq; single-cell	[54]
37434262	Spatially resolved transcriptomic profiles reveal unique defining molecular features of infiltrative 5ALA-metabolizing cells associated with glioblastoma recurrence	glioblastoma	Visium	ST-Pipeline; Seurat; SPATA2.0; GWmodel; hypeR	SPATA2.0-InferCNV	immunohistochemistry; single-cell	[78]
37187723	Single-nucleus and Spatially Resolved Intratumor Subtype Heterogeneity in Bladder Cancer	bladder cancer	Visium	Seurat; STUtility	NA	bulk-seq; single-nucleus	[138]
37564657	Integrative multi-omics analyses unravel the immunological implication and prognostic significance of CXCL12 in breast cancer	breast cancer	Visium	Seurat; SpaceRanger; STUtility	NA	immunohistochemistry; single-nucleus	[128]
37902941	TRPV1 inhibition suppresses non-small cell lung cancer progression by inhibiting tumour growth and enhancing the immune response	non-small cell lung cancer	PD	LoupeBrowser	NA	bulk-seq; immunohistochemistry; flow cytometry; mass spectrometry; cell culture	[135]
36816947	Spatially resolved transcriptomics revealed local invasion-related genes in colorectal cancer	colorectal cancer	Visium	SpaceRanger; STAT; SPOTlight; limma; monocle2	Seurat-FindVariableFeatures; Seurat-FindMarkers	immunohistochemistry; cell culture; single-cell	[69]
31931856	Identification and transfer of spatial transcriptomics signatures for cancer diagnosis	breast cancer	ST (pre-Visium)	ST-Pipeline; sklearn; DESeq2	Ward clustering	none	[27]
37127652	Spatial transcriptomic analysis of Sonic hedgehog medulloblastoma identifies that the loss of heterogeneity and promotion of differentiation underlies the response to CDK4/6 inhibition	medulloblastoma	Visium	SpaceRanger; stLearn; scanpy; Seurat; edgeR; clusterProfiler; Giotto; SingleR; scanpy	Louvain clustering; limma-voom; Giotto-PAGE	single-cell; cell culture	[63]
36215273	Spatial transcriptomic analysis delineates epithelial and	ependymoma	Visium	SpaceRanger; Seurat; Harmony; Clustree;	Seurat-CellCycleScoring	immunohistochemistry; bulk-	[66]

	mesenchymal subpopulations and transition stages in childhood ependymoma			LoupeBrowser; Slingshot; clustifyr		seq; cell culture; single-cell	
36703790	MIF promotes cell invasion by the LRP1-uPAR interaction in pancreatic cancer cells	pancreatic ductal adenocarcinoma	Visium	NA	NA	immunohistochemistry; flow cytometry; cell culture; bulk-seq	[131]
36125263	CD8+ and FoxP3+ T-Cell Cellular Density and Spatial Distribution After Programmed Death-Ligand 1 Check Point Inhibition	head and neck squamous cell carcinoma	GeoMx	NA	NA	immunohistochemistry; flow cytometry; cell culture; bulk-seq	[139]
36230778	Spatial Transcriptomic Analysis Reveals Associations between Genes and Cellular Topology in Breast and Prostate Cancers	breast cancer; prostate cancer	PD	gprofiler2; DEGAS	NA	single-cell	[136]
36641515	Clinical Significance of Signal Regulatory Protein Alpha (SIRPα) Expression in Hepatocellular Carcinoma	hepatocellular carcinoma	Visium	SpaceRanger; LoupeBrowser	NA	bulk-seq; immunohistochemistry	[130]
37811629	Spatial Heterogeneity of Integrins and Their Ligands in Primary Breast Tumors	breast cancer	Visium	SpaceRanger; Seurat; NICHES	Nearest Neighbor clustering; Seurat-FindAllMarkers	none	[71]
35906899	Quantification of spatial pharmacogene expression heterogeneity in breast tumors	breast cancer	Visium	SpaceRanger; LoupeBrowser	NA	none	[89]
34086429	Hypoxic microenvironment induced spatial transcriptome changes in pancreatic cancer	pancreatic ductal adenocarcinoma	Visium	SpaceRanger; Seurat	NA	bulk-seq; immunohistochemistry	[140]
36685583	The relevance between hypoxia-dependent spatial transcriptomics and the prognosis and efficacy of immunotherapy in claudin-low breast cancer	breast cancer	Visium	SpaceRanger; Seurat	NA	immunohistochemistry	[129]
37996891	Talniflumate abrogates mucin immune suppressive barrier improving efficacy of gemcitabine and nab-paclitaxel treatment in pancreatic cancer	pancreatic ductal adenocarcinoma	GeoMx	GeomxTools; Seurat; clusterProfiler	Seurat-AddModuleScore	cell culture; immunohistochemistry	[132]
36224014	Spatial Transcriptomics Analysis Reveals that CCL17 and CCL22 are Robust Indicators of a Suppressive Immune Environment in Angioimmunoblastic T Cell Lymphoma (AITL)	angioimmunoblastic T-cell lymphoma	Visium	SpaceRanger; scanpy; clusterProfiler; Seurat; Cell2location	Leiden clustering	immunohistochemistry; single-cell	[51]
35109839	Spatial maps of hepatocellular carcinoma transcriptomes highlight an unexplored landscape of heterogeneity and a novel gene signature for survival	hepatocellular carcinoma	Visium	SpaceRanger; Seurat	NA	bulk-seq	[98]

38235128	Integrative analysis of disulfidptosis and immune microenvironment in hepatocellular carcinoma: a putative model and immunotherapeutic strategies	hepatocellular carcinoma	Visium	Seurat; spacexr; SPOTlight; scMetabolism; stLearn	Seurat-SpatialDimPlot; hierarchical clustering	bulk-seq; single-cell; immunohistochemistry	[76]
35831529	Multidimensional Immunophenotyping of Intraductal Papillary Mucinous Neoplasms Reveals Novel T Cell and Macrophage Signature	intraductal papillary mucinous neoplasm	GeoMx	AtoMX	NanoString-SpatialDecon	flow cytometry; single-cell	[141]
36621024	Subclonal evolution and expansion of spatially distinct THY1-positive cells is associated with recurrence in glioblastoma	glioblastoma	Visium	Vslide; SpaceRanger; Seurat; MAGIC; clusterProfiler	Nearest-Neighbor clustering	cell culture; immunohistochemistry	[57]

Table S5. Overview of study aims and ST applications in analyzed articles. All references are included with the main article text.

PMID	Article Title	Study Aim	ST Application Purpose	Ref.
36708811	Identification of a tumour immune barrier in the HCC microenvironment that determines the efficacy of immunotherapy	discovering the specific structure of the tumor microenvironment (TME) and its role in immunotherapy efficacy in hepatocellular carcinoma	tumor heterogeneity; GSEA; DEA	[49]
36162205	Integrating microarray-based spatial transcriptomics and single-cell RNA-sequencing reveals tissue architecture in esophageal squamous cell carcinoma	discovering spatial composition of cells within the tumor microenvironment (TME) of esophageal carcinoma	tumor heterogeneity; MIA; DEA; cell-type deconvolution; KEGG; cell trajectory analysis	[53]
36967539	Spatiotemporally deciphering the mysterious mechanism of persistent HPV-induced malignant transition and immune remodelling from HPV-infected normal cervix, precancer to cervical cancer: Integrating single-cell RNA-sequencing and spatial transcriptome	describing the temporal transition and spatial distribution of cellular subsets during cervical carcinoma progression	tumor heterogeneity; DEA; cell-type deconvolution; GO; KEGG	[75]
37081869	Integrating single-cell RNA-seq and spatial transcriptomics reveals MDK-NCL dependent immunosuppressive environment in endometrial carcinoma	assessing the cell populations in the tumor microenvironment (TME) of endometrial carcinoma	tumor heterogeneity; GSVA	[118]
35794563	Spatial transcriptomics atlas reveals the crosstalk between cancer-associated fibroblasts and tumor microenvironment components in colorectal cancer	exploring the crosstalk between cancer-associated fibroblasts and microenvironment in the pathogenesis of colorectal cancer	tumor heterogeneity; ssGSEA; GSVA; KEGG; cell trajectory analysis	[119]
37263303	Spatially Resolved Multi-Omics Single-Cell Analyses Inform Mechanisms of Immune Dysfunction in Pancreatic Cancer	unveiling a detailed view of the immune micromilieu of pancreatic ductal adenocarcinoma	tumor heterogeneity; cell-type deconvolution; DEA	[67]
37488671	Integrated single-cell and spatial transcriptomic profiling reveals higher intratumour heterogeneity and epithelial-fibroblast interactions in recurrent bladder cancer	examining the tumor microenvironment (TME) characteristics between primary and recurrent bladder tumors	tumor heterogeneity; cell-type deconvolution; GSEA; DEA; WGCNA	[64]
36153593	Intratumor heterogeneity and T cell exhaustion in primary CNS lymphoma	in-depth characterization of primary central nervous system lymphoma (PCNSL)	expression distribution	[97]
35618288	Spatial transcriptomics of macrophage infiltration in non-small cell lung cancer reveals determinants of sensitivity and resistance to anti-PD1/PD-L1 antibodies	investigating the predictive value of tumor-associated macrophages (TAM) infiltration in patients with non-small cell lung cancer treated with immune checkpoint blockers	tumor heterogeneity; DEA	[134]
37125608	Primary Aldosteronism: Spatial Multiomics Mapping of Genotype-Dependent Heterogeneity and Tumor Expansion of Aldosterone-Producing Adenomas	deciphering the roles of transcriptome and metabolome reprogramming in tumor microenvironment (TME) pathogenesis of adrenocortical aldosterone-producing adenoma (APA) carrying a somatic mutation that drives aldosterone overproduction	tumor heterogeneity; GO; DEA; GSEA	[137]
37344903	Interactions between MFAP5 + fibroblasts and tumor-infiltrating myeloid cells shape the malignant microenvironment of colorectal cancer	discovering cell-cell interactions within the tumor microenvironment (TME)	tumor heterogeneity; ssGSEA; DEA	[133]

37723590	Spatial transcriptomics analysis of neoadjuvant cabozantinib and nivolumab in advanced hepatocellular carcinoma identifies independent mechanisms of resistance and recurrence	discovering the hepatocellular carcinoma heterogeneity in cellular signaling networks within the tumor microenvironment (TME) that underlie responses to the multi-tyrosine kinase inhibitor therapy	tumor heterogeneity; DEA; GSEA	[60]
38115703	Single-cell and spatial transcriptomics reveal POSTN(+) cancer-associated fibroblasts correlated with immune suppression and tumour progression in non-small cell lung cancer	examining the influence of cancer-associated fibroblasts (CAF) subpopulations on the non-small cell lung cancer (NSCLC) progression	tumor heterogeneity; cell-type deconvolution; DEA	[38]
37153569	Dissecting order amidst chaos of programmed cell deaths: construction of a diagnostic model for KIRC using transcriptomic information in blood-derived exosomes and single-cell multi-omics data in tumor microenvironment	constructing a diagnostic model depicting the landscape of programmed cell death (PCD)-associated genes in kidney renal clear cell carcinoma	predictive model; GO; KEGG; DEA	[25]
37951222	Artificial intelligence-based pathology as a biomarker of sensitivity to atezolizumab-bevacizumab in patients with hepatocellular carcinoma: a multicentre retrospective study	developing an artificial intelligence (AI) model able to estimate ABRS (atezolizumab-bevacizumab response signature) expression directly from histological slides of hepatocellular carcinoma	predictive model	[26]
38077210	Spatial architecture of high-grade glioma reveals tumor heterogeneity within distinct domains	interrogating the transcriptional landscape of high-grade glioblastoma	tumor heterogeneity; KEGG; cell-type deconvolution; ssGSEA; DEA	[88]
37124494	Spatial transcriptomics reveals the heterogeneity and FGG+CRP+ inflammatory cancer-associated fibroblasts replace islets in pancreatic ductal adenocarcinoma	understanding the spatial heterogeneity of the tumor microenvironment (TME) in pancreatic cancer (PC)	tumor heterogeneity; GO; GSEA	[58]
38050079	Integrative multiomics enhancer activity profiling identifies therapeutic vulnerabilities in cholangiocarcinoma of different etiologies	identifying potential therapeutic targets in different subtypes of cholangiocarcinoma (CCA)	tumor heterogeneity; GSEA; cell-type deconvoluion	[95]
37397378	Integration analysis of single-cell and spatial transcriptomics reveal the cellular heterogeneity landscape in glioblastoma and establish a polygenic risk model	discovering the relationship between cellular heterogeneity, tumor microenvironment, and glioblastoma progression	tumor heterogeneity; DEA; cell-type deconvolution; GSEA; cell trajectory analysis	[54]
37434262	Spatially resolved transcriptomic profiles reveal unique defining molecular features of infiltrative 5ALA-metabolizing cells associated with glioblastoma recurrence	understanding the 5-aminolevulinic acid-metabolizing cells biology as an early a priori proxy of glioblastoma (GBM) recurrence	tumor heterogeneity; WGCNA; CNV; DEA	[78]
37187723	Single-nucleus and Spatially Resolved Intratumor Subtype Heterogeneity in Bladder Cancer	investigating the extent and possible clinical impact of intratumor subtype heterogeneity across early and more advanced stages of bladder cancer	tumor heterogeneity	[138]
37564657	Integrative multi-omics analyses unravel the immunological implication and prognostic significance of CXCL12 in breast cancer	detecting the role of CXCL12 in breast cancer and exploring novel CXCL12-related biomarkers	tumor heterogeneity; WGCNA; ssGSEA	[128]
37902941	TRPV1 inhibition suppresses non-small cell lung cancer progression by inhibiting tumour growth and enhancing the immune response	exploring the effects of TRPV1 on the development of non-small cell lung cancer (NSCLC)	expression distribution	[135]
36816947	Spatially resolved transcriptomics revealed local invasion-related genes in colorectal cancer	revealing local invasion-related genes in colorectal cancer (CRC)	tumor heterogeneity; GSVA; cell trajectory analysis; DEA	[69]

31931856	Identification and transfer of spatial transcriptomics signatures for cancer diagnosis	distinguishing ductal carcinoma in situ (DCIS) from invasive ductal carcinoma (IDC) regions in clinical biopsies through automated tool	predictive model; DEA	[27]
37127652	Spatial transcriptomic analysis of Sonic hedgehog medulloblastoma identifies that the loss of heterogeneity and promotion of differentiation underlies the response to CDK4/6 inhibition	determining the spatial organization of cellular states underlying each medulloblastoma (MB) subgroup and how this impacts response to therapy	DEA; tumor heterogneity; GSEA; cell-type deconvolution	[63]
36215273	Spatial transcriptomic analysis delineates epithelial and mesenchymal subpopulations and transition stages in childhood ependymoma	understanding the tumor microenvironment (TME) of childhood brain tumor ependymoma	cell trajectory analysis; DEA; tumor heterogeneity; GSVA	[66]
36703790	MIF promotes cell invasion by the LRP1-uPAR interaction in pancreatic cancer cells	examining the role of macrophage migration inhibitory factor (MIF) in pancreatic ductal adenocarcinoma (PDAC)	expression distribution	[131]
36125263	CD8+ and FoxP3+ T-Cell Cellular Density and Spatial Distribution After Programmed Death-Ligand 1 Check Point Inhibition	analyzing CD8+ and FoxP3+ T-cell properties in head and neck squamous cell carcinoma (HNSCC) samples from a neoadjuvant trial of durvalumab +/- metformin	expression distribution; GSEA	[139]
36230778	Spatial Transcriptomic Analysis Reveals Associations between Genes and Cellular Topology in Breast and Prostate Cancers	determining how gene expression of cells is tied to their spatial relationships (i.e. topology) in breast and prostate cancers	GO; GSEA; tumor heterogeneity; KEGG	[136]
36641515	Clinical Significance of Signal Regulatory Protein Alpha (SIRPα) Expression in Hepatocellular Carcinoma	clarifying the clinical significance of signal regulatory protein alpha (SIRPα) expression in hepatocellular carcinoma (HCC)	expression distribution	[130]
37811629	Spatial Heterogeneity of Integrins and Their Ligands in Primary Breast Tumors	characterizing the morphological and spatial heterogeneity of primary breast tumors in the context of expression profiles of integrins and their ligands	tumor heterogoneity	[71]
35906899	Quantification of spatial pharmacogene expression heterogeneity in breast tumors	determining if selected pharmacogenes are expressed heterogeneously across breast cancer tissues and which pharmacogenes have the most spatial expression heterogeneity	tumor heterogoneity; GSEA	[89]
34086429	Hypoxic microenvironment induced spatial transcriptome changes in pancreatic cancer	understanding the spatial distribution of hypoxia-related heterogeneity in pancreatic ductal adenocarcinoma (PDAC)	tumor heterogeneity; GSEA; DEA	[140]
36685583	The relevance between hypoxia-dependent spatial transcriptomics and the prognosis and efficacy of immunotherapy in claudin-low breast cancer	examining the spatial overview of hypoxia-associated heterogeneity in claudin-low breast cancer	tumor heterogeneity; ssGSEA; DEA	[129]
37996891	Talniflumate abrogates mucin immune suppressive barrier improving efficacy of gemcitabine and nab-paclitaxel treatment in pancreatic cancer	identifying actionable pathways that underpin therapeutic resistance in the early stages of pancreatic ductal carcinoma (PDAC)	tumor heterogeneity; DEA; GSEA	[132]
36224014	Spatial Transcriptomics Analysis Reveals that CCL17 and CCL22 are Robust Indicators of a Suppressive Immune Environment in Angioimmunoblastic T Cell Lymphoma (AITL)	investigating the angioimmunoblastic T-cell lymphoma (AITL) tumor immune microenvironment	GSEA; DEA; NMF; cell-type deconvolution; tumor heterogeneity	[51]

35109839	Spatial maps of hepatocellular carcinoma transcriptomes highlight an unexplored landscape of heterogeneity and a novel gene signature for survival	delineating the molecular landscape of hepatocellular carcinoma (HCC)	DEA; GO; KEGG	[98]
38235128	Integrative analysis of disulfidptosis and immune microenvironment in hepatocellular carcinoma: a putative model and immunotherapeutic strategies	exploring the landscape of disulfidptosis and the immune microenvironment of hepatocarcinoma cells (HCC)	tumor heterogeneity; DEA; cell-type deconvolution; WGCNA; GSVA	[76]
35831529	Multidimensional Immunophenotyping of Intraductal Papillary Mucinous Neoplasms Reveals Novel T Cell and Macrophage Signature	profiling immune cell infiltration composition in dysplastic progression of intraductal papillary mucinous neoplasms (IPMN)	DEA; expression distribution; cell-type deconvolution	[141]
36621024	Subclonal evolution and expansion of spatially distinct THY1-positive cells is associated with recurrence in glioblastoma	investigating the molecular pathways mediating glioblastoma resistance, with the goal of identifying novel therapeutic opportunities	GSEA; tumor heterogeneity	[57]
